# Long non‐coding RNA HULC stimulates the epithelial–mesenchymal transition process and vasculogenic mimicry in human glioblastoma

**DOI:** 10.1002/cam4.4083

**Published:** 2021-07-02

**Authors:** Tiantian Yin, Jing Wu, Yuchen Hu, Min Zhang, Jie He

**Affiliations:** ^1^ Clinical Pathology Center The First Affiliated Hospital of USTC Division of Life Sciences and Medicine University of Science and Technology of China Hefei Anhui People’s Republic of China

**Keywords:** EMT, GBM, invasion, LncRNA HULC, VM

## Abstract

**Background:**

Long non‐coding RNA (lncRNA) HULC (highly upregulated in liver cancer) is considered as an oncogenic factor for various malignant tumors. This study aimed to reveal the role of lncRNA HULC in the malignant behavior of glioblastoma (GBM) by exploring its effects on the epithelial–mesenchymal transition (EMT) and vasculogenic mimicry (VM) of human GBM.

**Materials and Methods:**

The contents of VM in 27 GBM samples were assessed by immunohistochemistry‐histology and their association with progress‐free survival (PFS) was analyzed. Human GBM SHG44 and U87 cells were manipulated to establish stable lncRNA HULC overexpressing and silencing cells by lentivirus‐based technology. The effects of altered lncRNA HULC on vasculogenic tubular formation, invasion, and EMT process in GBM cells were tested in vitro and the growth of implanted GBM tumors and their EMT process were examined in vivo.

**Results:**

The numbers of VM were positively associated with disease progression, but negatively with PFS periods of GBM patients. Compared with the control vec cells, lncRNA HULC overexpression significantly increased the tubular formation, invasion, and EMT process of both SHG44 and U87 cells, accompanied by promoting the growth of implanted GBM tumors and EMT process in mice. LncRNA HULC silencing had opposite effects on the tubular formation, invasion, and EMT process as well as tumor growth of GBM cells.

**Conclusion:**

LncRNA HULC stimulates the EMT process and VM in human GBM, and may be a therapeutic target for intervention of GBM.

## Graphical abstract

LncRNA HULC may increase VM to promote the progression of glioblastoma (GBM) by enhancing the epithelial–mesenchymal transition process in GBM. Our findings may uncover a therapeutic target and help in understanding the molecular pathogenesis of GBM.

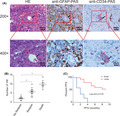

## 1 INTRODUCTION

Glioblastoma (GBM) is the most common type of primary malignant tumor in human brain with poor prognosis. GBM is the most malignant grade IV of glioma, according to the 2016 WHO Central Nervous System Tumor Classification. Approximately, more than 90% of patients with GBM have a survival period of less than 14 months after diagnosis. Traditional therapeutic strategies for GBM include surgical resection, radiotherapy, and chemotherapy, as well as immunotherapies. However, the efficacy of these therapies is limited as these therapies do not prolong the survival of GBM patients.^1^ The lack of effective therapy is largely attributed to our poor understanding of the molecular pathogenesis of GBM. Hence, understanding the pathogenesis of GBM will be of high significance in discovery of new therapeutic targets and strategies for GBM.

The rapid growth of GBM cells in the brain depends on sufficient blood supply of nutrients and oxygen, which are critical for the proliferation and invasion of GBM cells. It is well known that tumor cells can form microvascular channels, vasculogenic mimicry (VM), to increase nutrient and blood supply in different types of tumors, including GBM.^2^ VM is an environment‐adaptive behavior of tumor cells, and made by highly malignant and low‐differentiated tumor cells, such as GBM cells, displaying the potential stem cell characteristics. The tumor cells mimic vascular endothelial cells to construct microvascular channels to support the growth of tumors.^3^ Tumor cells that participate in the process of VM usually undergo the epithelial–mesenchymal transition (EMT) process, fascinating their migration, and invasion. They exhibit mesenchymal cell characters with upregulated N‐cadherin, vimentin, Snail and Slug expression, promoting matrix metalloproteinase (MMP) expression, but downregulated E‐cadherin expression. As a result, tumor cells lose their polarity and increase their mobility while weakening cell adhesion, leading to invasion.^4^ However, little is known on how the EMT and VM are regulated in GBM.

Long non‐coding RNA (lncRNA) is a class of non‐coding RNAs and can regulate many biological processes. LncRNA HULC (highly upregulated in liver cancer) was initially detected in liver cancer, and is also abnormally expressed in GBM.^5^ However, there is no information on whether and how lncRNA HULC can regulate the development and progression of human GBM. Given that VM and EMT processes are crucial for the proliferation and metastasis of malignant tumor cells we hypothesize that lncRNA HULC may promote the growth of human GBM and enhance the EMT process and VM of GBM cells.^6^

In this study, we quantified the numbers of VM in GBM tissues and explored the potential roles of lncRNA HULC in regulating the tubular formation, invasion, and EMT process of human GBM cells in vitro and in vivo.

## 2 MATERIALS AND METHODS

### 2.1 Patients and tissue specimens

A total of 27 GBM patients were recruited in Department of Neurosurgery, The First Affiliated Hospital of USTC between September 2019 and December 2019. Those GBM patients were diagnosed by clinical symptoms and radiological examination, and their grades were evaluated, according to the 2016 WHO Central Nervous System Tumor Classification. All of the patients underwent surgical resection of the tumor and their surgical tumor specimens were collected. Their demographic and clinicopathological data were collected and shown in Table 1. Those patients were followed up until July 2020. Their overall survival and progress‐free survival (PFS) were calculated. The tumor tissue sections (3 µm) from each specimen were stained with hematoxylin‐eosin and reviewed by two pathologists.

**TABLE 1:** The demographic and clinical data of GBM patients

Case	Age (years)	Sex	Tumor site	PFS (M)	OS (M)	Number of VM
1	72	Male	Left triangle	2	6	14
2	53	Male	Left frontal lobe	No relapse	Alive	2
3	67	Male	Right frontal lobe	3	6	10
4	55	Male	Left thalamus	1	Alive	7
5	60	Male	Right parietal and occipital lobe	8	Alive	5
6	13	Female	Central brain area	No relapse	Alive	2
7	41	Female	Right temporal lobe	2	Alive	10
8	13	Female	Right frontal and temporal lobe	4	Alive	7
9	38	Female	Brain	3	Alive	3
10	57	Male	Left insula and basal ganglia	3	Alive	4
11	50	Male	Corpus callosum	2	Alive	6
12	67	Female	Right frontal lobe	No relapse	Alive	3
13	52	Female	Right temporal lobe	No relapse	Alive	4
14	70	Male	Right temporal lobe	4	6	7
15	23	Male	Frontal and temporal lobe	No relapse	Alive	1
16	48	Male	Corpus callosum	1	1	8
17	54	Male	Left temporal lobe	5	Alive	5
18	53	Male	Right occipital lobe	2	3	12
19	78	Male	Left frontal lobe	1	1	9
20	50	Male	Right temporal lobe	No relapse	Alive	3
21	50	Female	Left frontal lobe	3	Alive	8
22	54	Male	Right thalamus	6	Alive	3
23	63	Male	Right temporal lobe	5	Alive	6
24	47	Female	Right frontal lobe	No relapse	Alive	2
25	52	Male	Right lateral ventricle	1	1	11
26	61	Male	Right triangle	No relapse	Alive	1
27	64	Male	Left insula and basal ganglia	1	5	9

### 2.2 Cell culture and lentivirus transduction

Human GBM SHG44 and U87 cells were obtained from the China Centre for Type Culture Collection (Wuhan, China) and identified by short tandem repeat. The cells were cultured in DMEM medium containing 10% fetal bovine serum (Biological Industries). SHG44 and U87 cells were transduced with control vehicle lentivirus or experimental lentivirus to generate control (vec), stable HULC overexpression (HULC), control silencing (si‐NC), and HULC silencing cells (HULC‐siRNA) using specific lentiviruses generated by our research group previously.^5^

### 2.3 Matrigel tube formation assay

The different groups of cells (6 × 10^4^ cells/well) were cultured in triplicate in 24‐well plates that were pre‐coated with Matrigel (300 μl/well; BD Bioscience) at 37℃ for 4 h. The formed tubes were captured by photoimaging under a light microscope (Olympus) and the numbers of tubular structures in five random fields of each well were quantified using the Image J software in a blinded manner.

### 2.4 Transwell invasion assay

The impact of altered lncRNA HULC expression on cell invasion was tested in a transwell invasion assay using 24‐well transwell plates (8‐µm pore size; Corning). The different groups of cells (5 × 10^4^ cells/well) were cultured in triplicate in the upper chamber that had been coated with matrigel and complete DMEM (600 µl/well) was added to the lower chamber. One day later, the cells that invaded on the bottom surface of the upper chamber membrane were fixed with 4% paraformaldehyde and stained with 0.1% crystal violet. The stained cells in five fields randomly selected in each well were visualized and counted under a light microscope.

### 2.5 Quantitative RT‐PCR

Total RNA was extracted from individual groups of cells using Total RNA Kit (Qiagen). After quantification of RNA concentrations using the Nanodrop spectrophotometer (ND‐100; Thermo Scientific), the RNA samples were reversely transcribed into cDNA. The relative levels of LncRNA HULC, E‐cadherin, MMP2, MMP9, N‐cadherin, Slug, Snail, and Vimentin to the control GAPDH were quantified in triplicate by qRT‐PCR using the specific primers (Table 2), the SYBR Premix Ex Taq and Taqman Universal Master Mix II (Applied Biosystems). The data were analyzed by the 2^−ΔΔCt^ method.

**TABLE 2:** The primer sequences for qRT‐PCR

Gene	Sequence
LncRNA HULC forward	5′‐CGCGTCACAGTGAACCGGT‐3′
LncRNA HULC reverse	5′‐AGTGCAGGGTCCGAGGTATT‐3′
E‐cadherin forward	5′‐ GCAGGTCTCCTCTTGGCTCT‐3′
E‐cadherin reverse	5′‐GTCGACCGGTGCAATCTTCA‐3′
MMP2 forward	5′‐CTGTAGAAAGAGCCCTGAAGAATC‐3′
MMP2 reverse	5′‐TGCCTTGCACATAGAAAGCAC‐3′
MMP9 forward	5′‐TGCTCTTCCCTGGAGACCTGA‐3′
MMP9 reverse	5′‐CTGCCACCCGAGTGTAACCA‐3′
N‐cadherin forward	5′‐CGGAGATCCTACTGGACGGT‐3′
N‐cadherin reverse	5′‐GGTTTGACCACGGTGACTAACC‐3′
Slug forward	5′‐ACTGTGTGGACTACCGCTGC‐3′
Slug reverse	5′‐AGGAGGTGTCAGATGGAGGAGG‐3′
Snail forward	5′‐CCTGTCTGCGTGGGTTTTTG‐3′
Snail reverse	5′‐CCAGTGAGTCTGTCAGCCTTTGT‐3′
Vimentin forward	5′‐CTGGATTCACTCCCTCTGGTT‐3′
Vimentin reverse	5′‐TCGTGATGCTGAGAAGTTTCGTT‐3′
GAPDH forward	5′‐GAACGGGAAGCTCACTGG‐3′
GAPDH reverse	5′‐GCCTGCTTCACCACCTTCT‐3′

### 2.6 Western blot

The different groups of cells were harvested and lysed in RIPA buffer, followed by centrifugation. After quantification of protein concentrations using a BCA protein assay kit, individual cell lysates (30 µg/lane) were separated by sodium dodecyl sulfate–polyacrylamide gel electrophoresis (SDS–PAGE) on 10% gels, and transferred onto polyvinylidene difluoride membranes (Millipore). The membranes were blocked with 5% fat‐free dry milk in TBST and probed with primary antibodies against E‐cadherin (4A2, 1:500), MMP2 (D4M2N, 1:1000), MMP9 (D6O3H, 1:500), N‐cadherin (D4R1H, 1:500), Slug (C19G7, 1:1000), Snail (C15D3, 1:500), Vimentin (D21H3, 1:1000), and GAPDH (D16H11, 1:1000; Cell Signaling Technology). The bound antibodies were detected with horseradish peroxidase (HRP)‐conjugated secondary antibody (Cell Signaling Technology) and visualized using the enhanced chemiluminescence kit. The data were analyzed using Image J software.

### 2.7 In vivo experiments

An orthotopic GBM xenograft mouse model was established using the different groups of U87 cells as described previously.^5^ Briefly, BALB/c nude mice at 8 weeks of age were randomized and intracranial implanted with U87‐vec, U87‐HULC, U87‐siNC, or U87‐HULC‐siRNA (*n* = 7 per group). The mice were monitored for their body weights and behaviors. When cachexia occurred, they were euthanized by CO_2_ inhalation, and their brains were dissected. Their tumors were dissected and weighed. The survival of remaining tumor bearing mice was monitored up to 45 days post implantation. The animal experiments were conducted in strict accordance with the Guidelines for the Care and Use of Laboratory Animals, 8th edition, issued by the National Institutes of Health.

### 2.8 Immunohistochemistry

Human GBM tissue sections (3 µm) were routine‐stained with anti‐CD34 (10C9, ready‐to use; ZSGB‐BIO) or anti‐GFAP (L50‐823, ready‐to‐use; Maxim). The sections were further stained with periodic acid–Schiff (PAS) and counterstained with hematoxylin. Subsequently, the bound antibodies were detected with HRP‐ or alkaline phophatase (AP)‐multimer staining and visualized with DAB using the Opti View DAB IHC Detection Kit, Ultra View Universal DAB Detection Kit, Ultra View Universal Alkaline Phophatase Red Detection Kit, Bluing Reagent (Ventana Medical Systems) in a VENTANA Benchmark Ultra automatic staining machine.

The consecutive GBM sections (3 µm) from individual mice were subjected to immunohistochemistry using primary antibodies against E‐cadherin (4A2, 1:100), N‐cadherin (D4R1H, 1:100; Cell Signaling Technology), MMP2 (ab97779, 1:100), MMP9 (EPR22140‐154, 1:100), Slug +Snail (ab180714, 1:1000), Vimentin (EPR3776, 1:250), and HRP‐multimer staining. The images were semi‐quantified by two pathologists in a blinded manner, as previously described.^5^ Briefly, the antibody staining intensity was scored as 1: weakly positive/equivocal staining within >10% of the tumor cells; 2: moderately positive within >10% of the tumor cells or strong staining within ≤10% of the tumor cells; 3: strongly positive in >10% of the tumor cells. A total of 500 tumor cells selected randomly from each sample were evaluated.

### 2.9 Statistical analysis

Data are present as representative images or mean ± SEM. The difference between groups was analyzed using Student's *t*‐test, one‐way ANOVA, and Fisher's test where applicable and the survival data were analyzed by log rank test using GraphPad Prism 7 software (GraphPad). A *p*‐value of <0.05 was considered statistically significant.

## 3 RESULTS

### 3.1 Increased numbers of VM are associated with a poor prognosis of GBM patients

**FIGURE 1:**
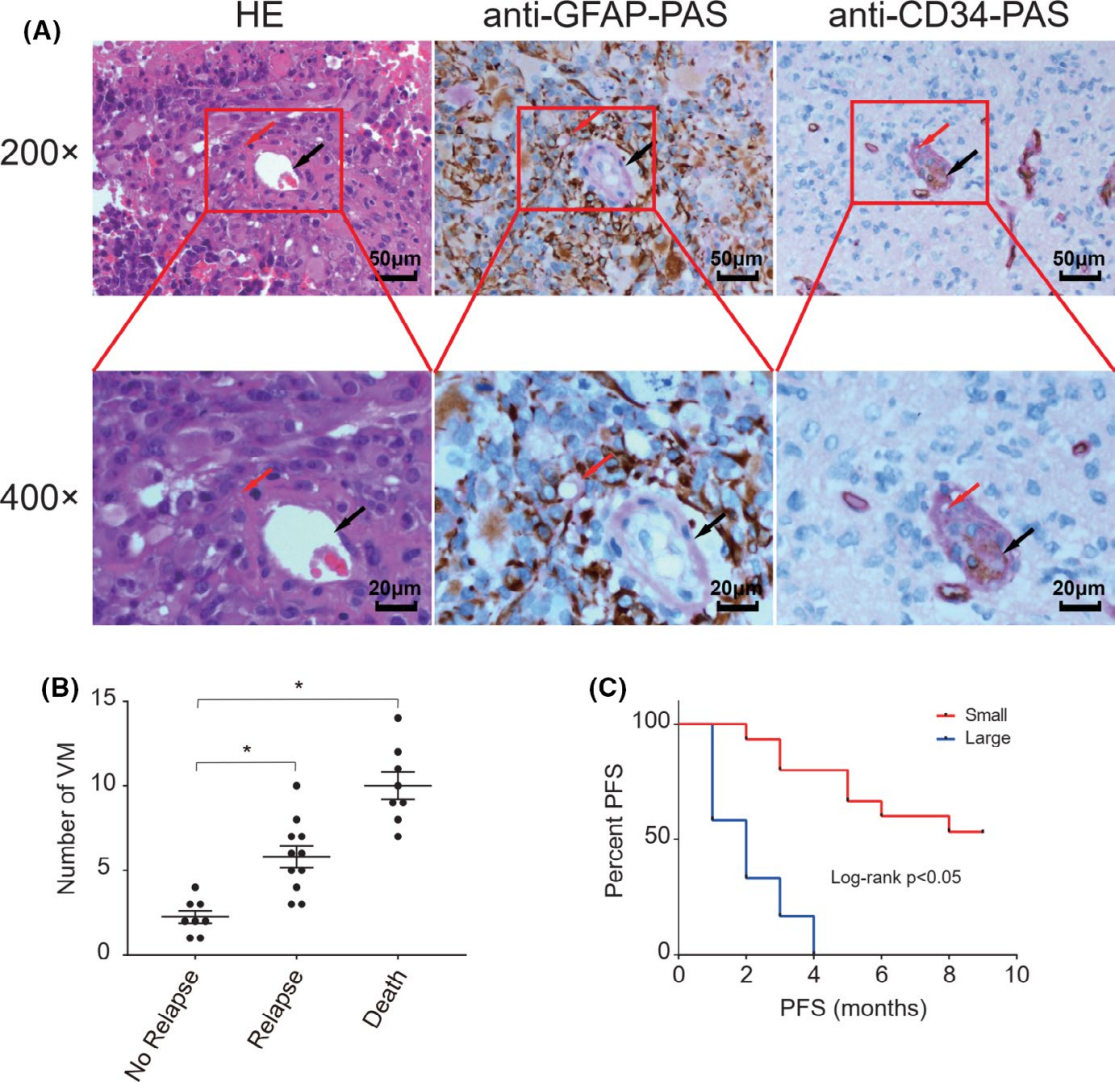
Increased numbers of vasculogenic mimicry (VM) are associated with the progression and poor progress‐free survival of glioblastoma (GBM) patients. The numbers of VM in 27 GBM specimens were examined by immunohistochemistry and histology by anti‐CD34 or anti‐GFAP and subsequent PAS staining. Those patients were stratified, according to relapse and death and the numbers of VM in GBM tissues of each group of GBM patients were analyzed. (A) Immunohistochemistry‐histological examination of VM in human GBM tissues (magnification, 200×, scale bar, 50 µm; magnification, 400×, scale bar, 20 µm). The VM is pointed by a red arrow. Blood vessels are shown by black arrows. Anti‐GFAP‐PAS or anti‐CD34‐PAS: The sections were first stained with anti‐GFAP or anti‐CD34 and subsequently with PAS. (B) Quantitative analysis of VM numbers in each group of patients. *p < 0.05. (C) The PFS of each group of patients. Small: the numbers of VM were less <7; Large: the numbers were ≥7 VM. *P *< 0.05 by Log rank test

To explore the potential role of VM in the progression of GBM, we examined the levels of VM in 27 GBM specimens by immunohistochemistry and histology using anti‐CD34‐PAS and anti‐GFAP‐PAS staining. As shown in Figure 1, vascular‐like structures were detected within the tumors and they were GFAP+, but CD34‐, suggesting that they were formed by tumor cells, but not from vascular endothelial cells. Quantification of the number of VM in stratified groups indicated that the numbers of VM in the patients with relapsed GBM were significantly greater than those with non‐relapsed GBM (*p *< 0.05), but were significantly less than those died from GBM in this population (*p *< 0.05, Figure 1B). Follow‐up of those patients revealed that GBM patients with less VM had a significantly longer PFS than those with more VM in this population (*p *< 0.05, Figure 1C). Hence, increased numbers of VM were associated with poor prognosis of GBM.

### 3.2 LncRNA HULC promotes the tube formation and invasion of human GBM cells in vitro

**FIGURE 2:**
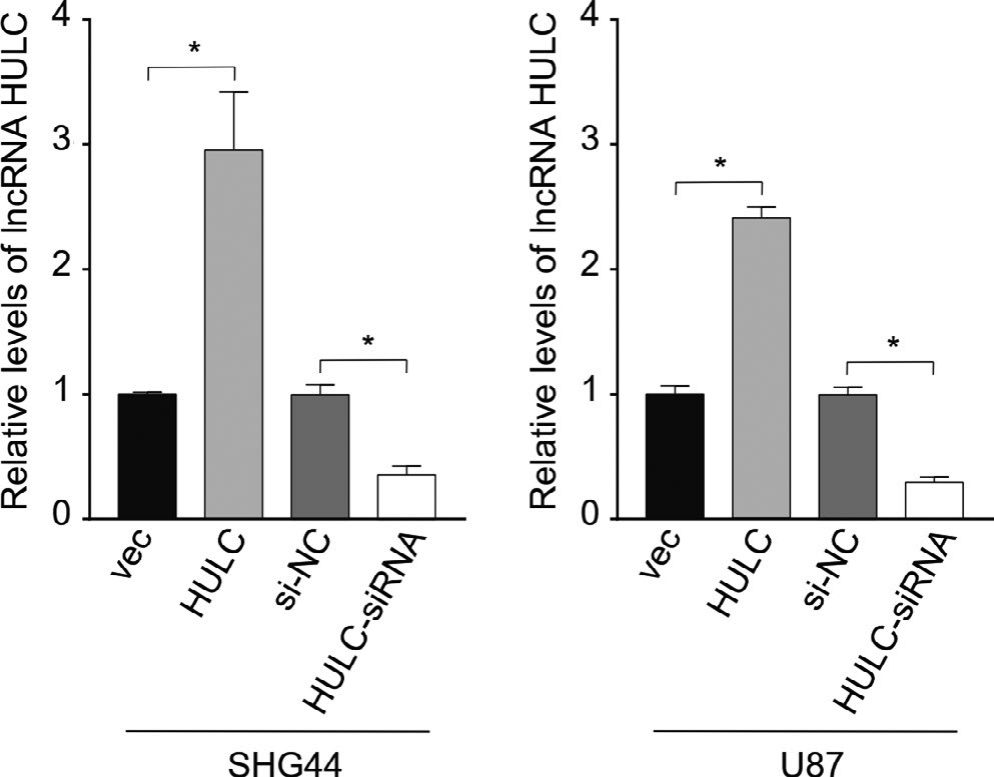
Quantitative RT‐PCR analysis of lncRNA HULC transcripts in different groups of cells. Human GBM SHG44 and U87 cells were transduced with vec lentivirus, lentivirus for lncRNA HULC overexpression (HULC), silencing (HULC‐siRNA), or control siRNA (siNC). The relative levels lncRNA HULC transcripts to control GAPDH in the stable HULC overexpression or silencing cells as well as their controls were determined by qRT‐PCR. Data are mean ± SEM of each group from two separate experiments. *p < 0.05 by Student *t*‐test

LncRNA HULC expression is upregulated malignant tumors, including GBM. To explore the role of lncRNA HULC in the VM formation and metastasis, human GBM SHG44 and U87 cells were transduced with different lentiviruses to generate stable HULC overexpressing and silencing cells and their lncRNA HULC expression was quantified by qRT‐PCR (Figure 2). The results indicated that the relative levels of lncRNA HULC transcripts in the SHG44‐HULC increased by near threefold, compared with that in the SHG44‐vec control cells while the lncRNA HULC transcripts in the SHG44‐HULC‐siRNA cells decreased by about 75%, related to that in the SHG44‐siNC cells. A similar pattern of lncRNA HULC transcripts was detected the different groups of U87 cells. Such data demonstrated a high efficacy in lncRNA HULC overexpression and silencing in these cells.

**FIGURE 3:**
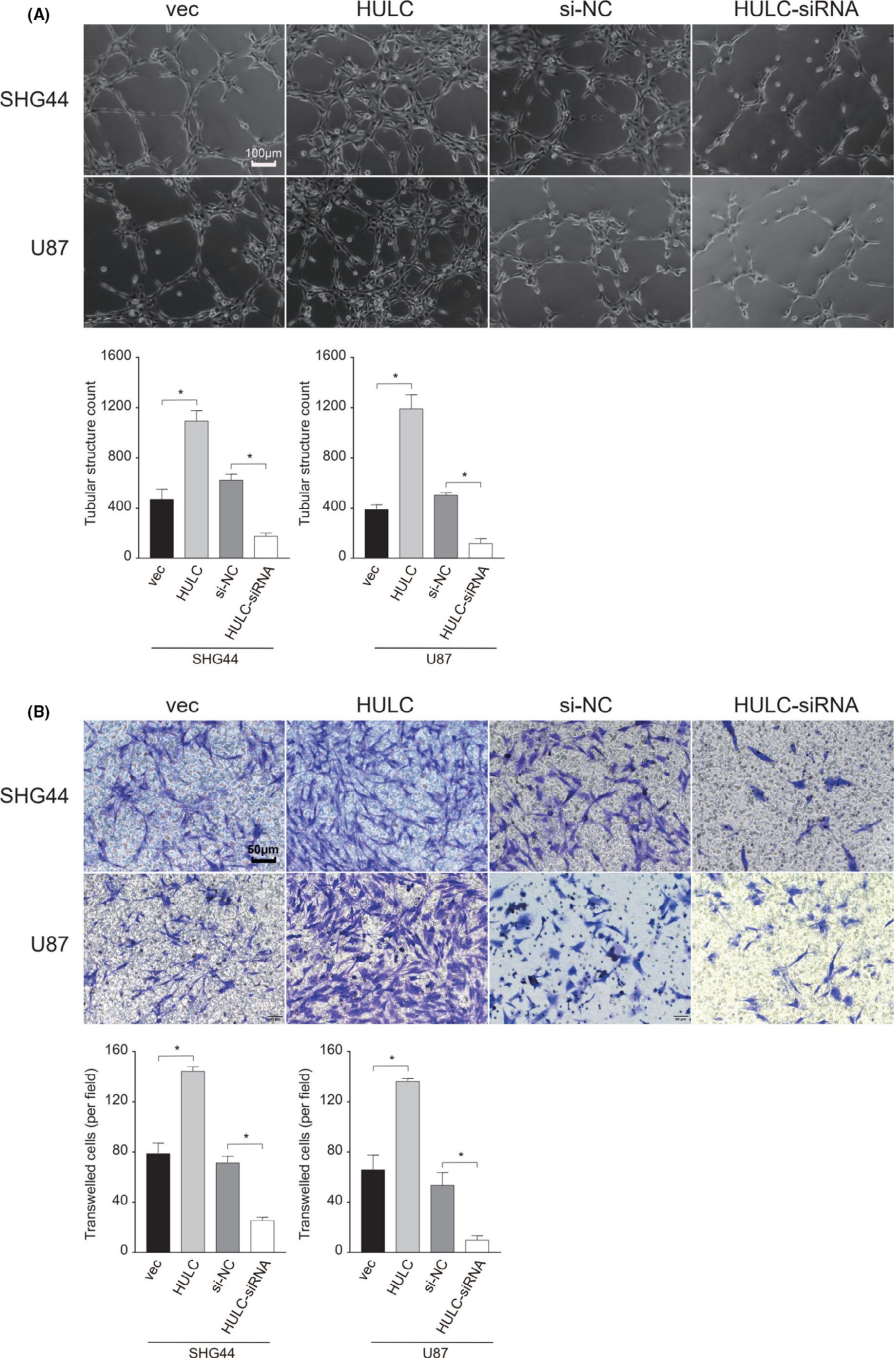
Altered lncRNA HULC expression modulates the tubular formation and invasion in the different groups of glioblastoma (GBM) cells. The different groups of cells were cultured on Matrigel and the tubular structures were measured in a blinded manner. Similarly, the invasion of different groups of cells was determined by transwell invasion assays. Data are representative images or expressed as mean ± SEM of each group from two separate experiments. (A) Photoimaging of VM tube formation in the cultured GBM cells in vitro (magnification, 100×; scale bar, 100 µm). (B) Invasion was measured by transwell assay (magnification, 200×; scale bar, 50 µm). *p<0.05 by Student *t*‐test

Next, we tested the impact of altered lncRNA HULC expression on the tube formation and invasion of GBM cells by the tube formation and transwell invasion assays. As shown in Figure 3A, in comparison with the U87‐vec or U87‐siNC controls, significantly increased numbers of tubes were detected in U87‐HULC cells and decreased numbers of tubes were observed in U87‐HULC‐siRNA cells. Similar results were obtained from the different groups of SHG44 cells (Figure 3A). These data clearly indicated that lncRNA HULC promoted the tube formation of GBM cells. Transwell invasion assays revealed that lncRNA HULC overexpression enhanced the invasion of GBM cells while its silencing reduced the numbers of invaded GBM cells, relative to that in the controls (Figure 3B). Such two independent lines of evidence demonstrated that lncRNA HULC promoted the tube formation and invasion of human GBM cell in vitro.

### 3.3 LncRNA HULC upregulates the expression of EMT‐related regulatory genes in GBM cells

**FIGURE 4:**
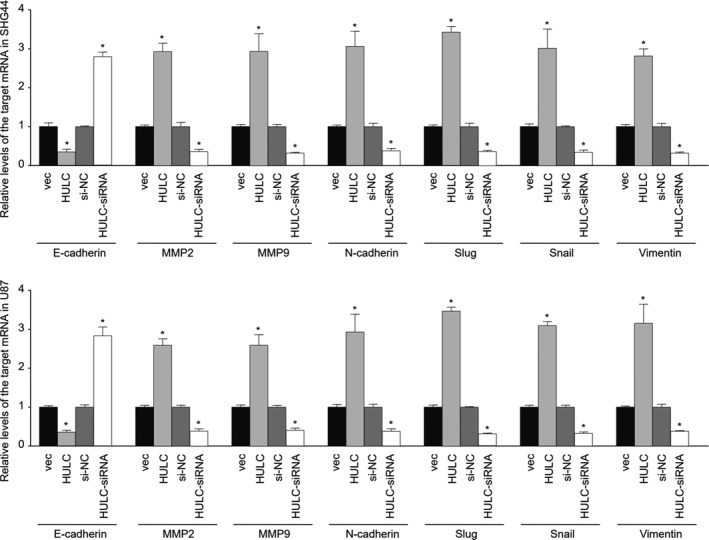
LncRNA HULC promotes the EMT process and MMP expression in GBM cells in vitro. The relative levels of E‐cadherin, N‐cadherin, Snail, Slug Vimentin, MMP2, and MMP9 expression to the control GAPDH mRNA transcripts in the different groups of GBM cells were determined by qRT‐PCR. Data are mean ± SEM of each group from two separate experiments. *p<0.05

**FIGURE 5:**
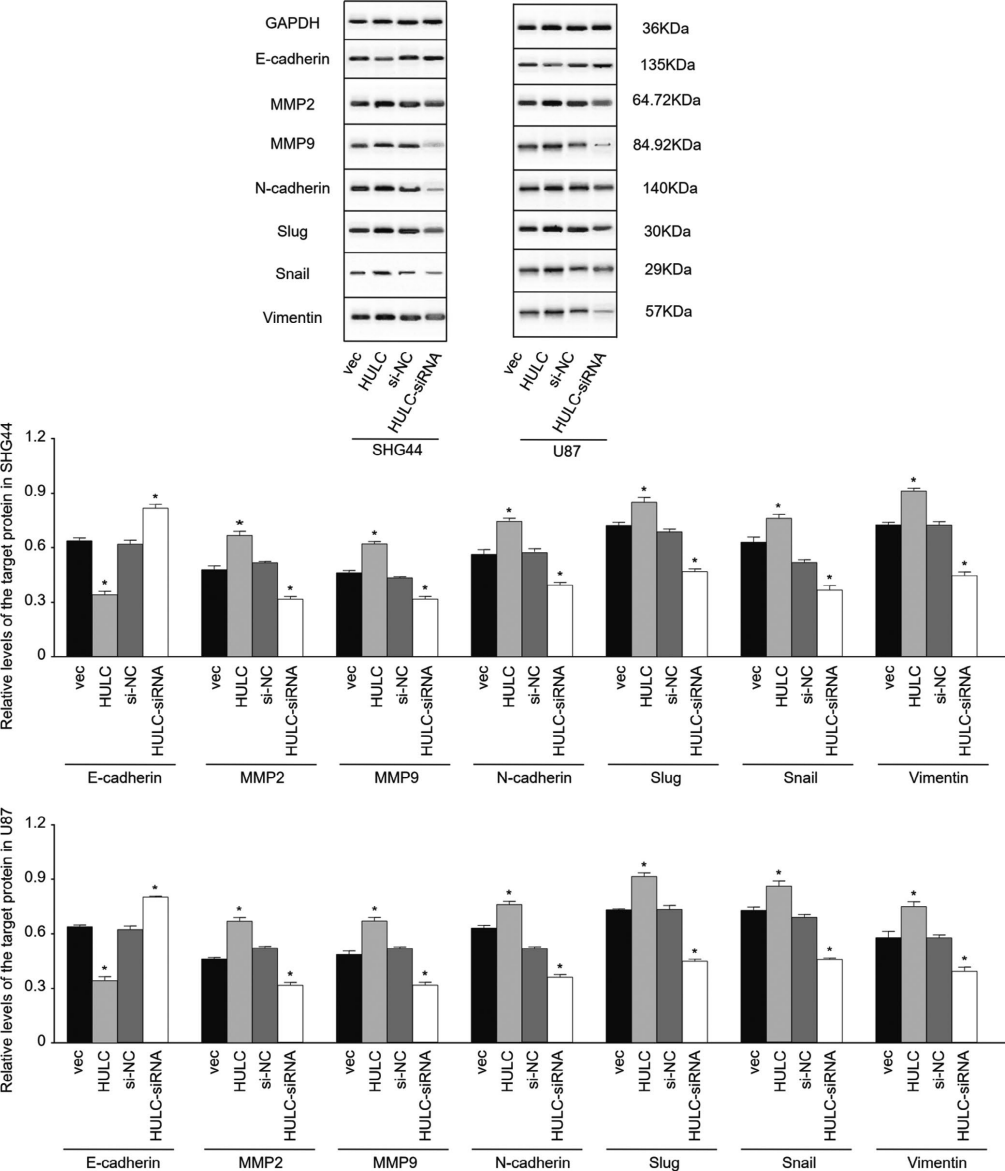
LncRNA HULC enhances the expression of EMT‐related molecules and regulators in GBM cells. The relative levels of E‐cadherin, N‐cadherin, Snail, Slug, vimentin, MMP2, and MMP9 to the control GAPDH expression in the different groups of GBM cells were determined by western blot. Data are representative images or expressed as mean ± SEM of each group from two separate. *p < 0.05

The EMT process is associated with VM and invasion of tumor cells. To understand how lncRNA HULC regulates the VM and invasion of GBM cells, we quantified the relative levels of EMT‐related regulatory gene expression in the different groups of cells by qRT‐PCR and western blot assays. In comparison with that in the SHG44‐vec or SHG44‐siNC control cells, significantly decreased levels of E‐cadherin mRNA transcripts were detected in SHG44‐HULC cells, but increased levels of them were observed in the SHG44‐HULC‐siRNA cells (Figure 4). In contrast, HULC overexpression significantly increased the relative levels of MMP2, MMP9, N‐cadherin, Slug, Snail, and Vimentin mRNA transcripts while HULC silencing decreased their levels in SHG44 cells. Similar patterns of their mRNA transcripts were detected in the different groups of U87 cells. Furthermore, similar data were obtained from the different groups of SHG44 and U87 cells by western blot assays (Figure 5). Collectively, the changes in the profiles of EMT‐related regulatory gene expression by altered lncRNA HULC indicated that lncRNA HULC promoted the EMT process of human GBM cells in vitro.

### 3.4 LncRNA HULC enhances the growth of xenograft GBM in mice, associated with promoting the EMT process

**FIGURE 6:**
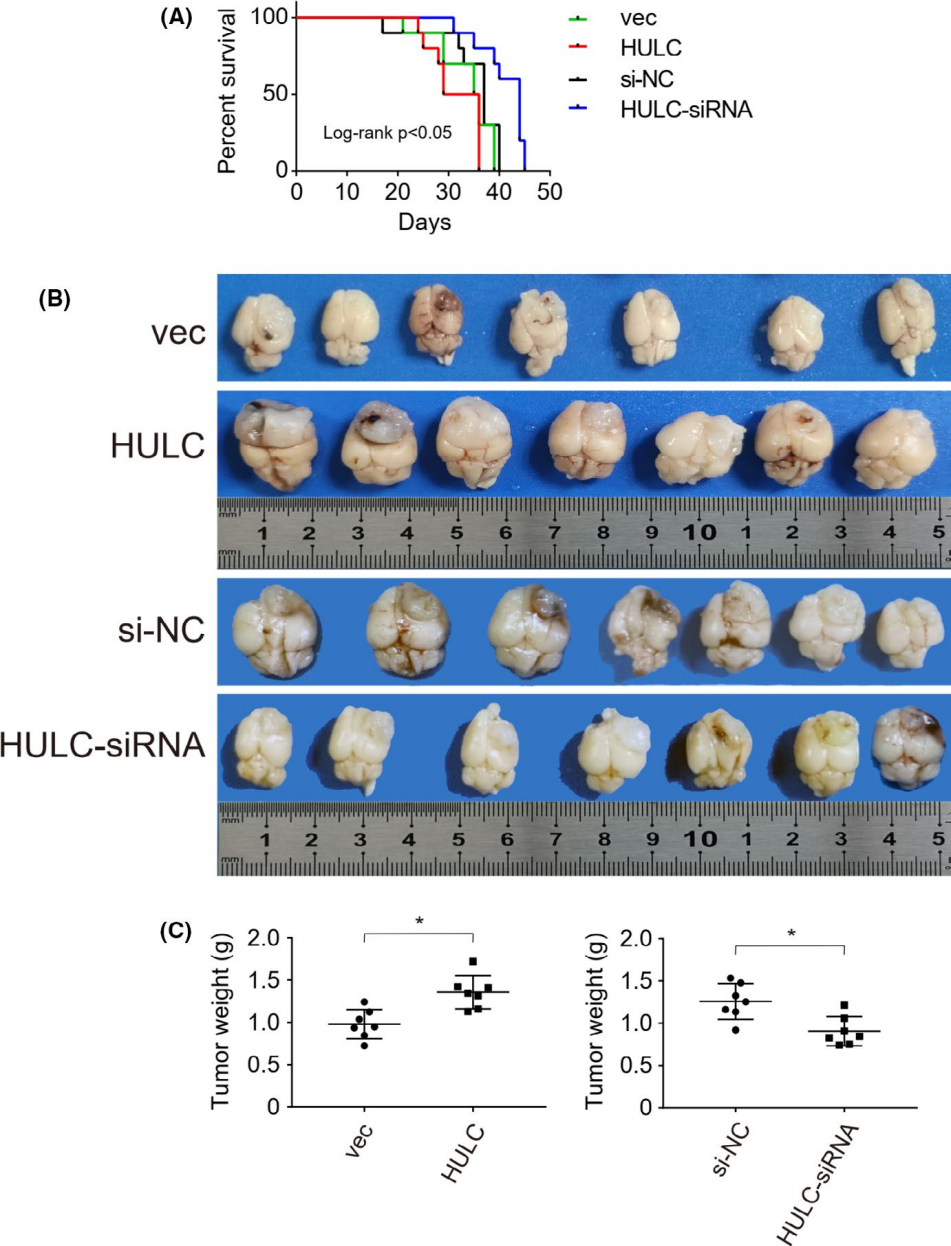
LncRNA HULC promotes the growth of implanted glioblastoma (GBM) in mice. BALB/c nude mice were implanted the indicated types of U87 cells in their brains and their survival was monitored. Their brains were dissected out for imaging and their tumor weights were measured. Data are their brain images, survival curves, and mean tumor weights in each group (*n *= 7 per group). (A) LncRNA HULC overexpression promoted the death of GBM‐bearing mice. (B) The brain images. (C) The tumor weights. *p < 0.05

To explore the role of lncRNA HULC in the growth of GBM, we tested the impact of altered lncRNA HULC expression on the growth of implanted GBM in mice. Individual BALB/c nude mice were inoculated orthotopically with control U87‐vec, U87‐HULC, U87‐siNC, or U87‐HULC‐siRNA GBM cells in their brains. Their survival was monitored up to 45 days post implantation. As shown in Figure 6A, the survival time periods of the HULC group of mice were significantly shorter than that of the vec control group (*p *< 0.05). Furthermore, the dissected brain tumor weights in the HULC group of mice were greater than that of the vec group (*p *< 0.05, Figure 6B,C). In contrast, the survival time periods and tumor weights in the HULC‐siRNA group of mice were longer and less than that in the control si‐NC group, respectively (Figure 6). Such data indicated the lncRNA HULC promoted the growth of implanted human GBM tumors in mice.

**FIGURE 7:**
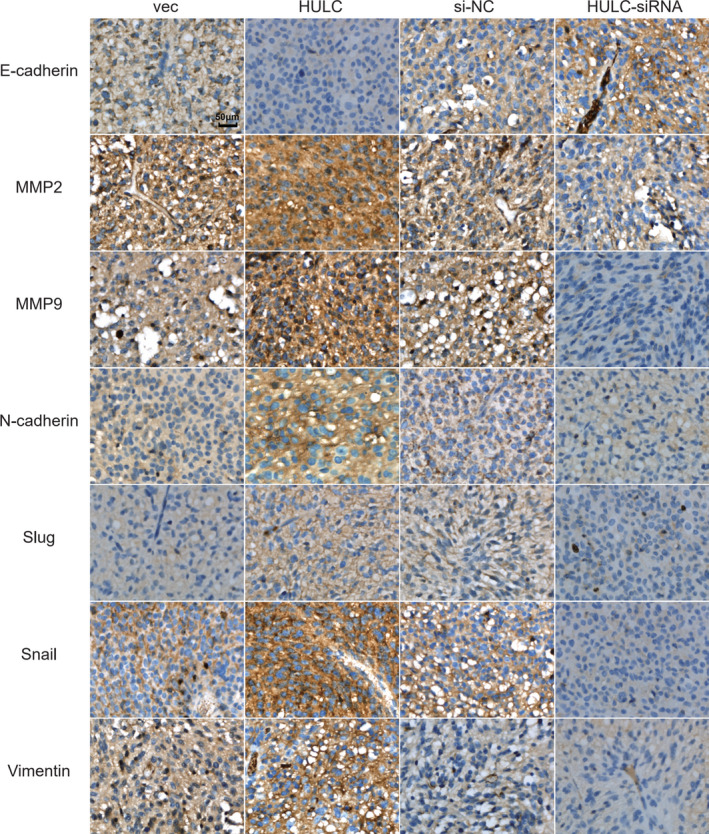
Immunohistochemistry analysis of EMT‐related molecule and regulator expression in glioblastoma (GBM) tumors. The expression of E‐cadherin, N‐cadherin, MMP2, MMP9, Snail, Slug, and vimentin in individual GBM tumors of different groups of mice was characterized by immunohistochemistry. Data are representative images of each group (*n *= 7 per group) of mice from three separate experiments (magnification, 200×; scale bar, 50 µm)

Immunohistochemistry analysis displayed that lncRNA HULC overexpression enhanced the EMT process in the GBM tumors, which were abrogated by lncRNA HULC silencing in GBM cells. Evidently, compared with the vec control tumors, we detected obviously lower levels of E‐cadherin, but higher levels of N‐cadherin, MMP2, MMP9, Slug, Snail, and Vimentin expression in the HULC group of tumors (Figure 7). Conversely, we observed higher E‐cadherin, but lower levels of N‐cadherin, MMP2, MMP9, Slug, Snail, and Vimentin expression in the HULC‐si group of tumors, relative to that in the si‐NC group of tumors (Figure 7). Together, such findings demonstrated that lncRNA HULC promoted the EMT process of human GBM tumors in mice.

## 4 DISCUSSION

It is well known that VM is crucial for the progression of malignant tumors by providing nutrients and eliminating metabolites.^7^ In this study we explored the role of VM in human GBM by the anti‐CD34‐PAS and anti‐GFAP‐PAS staining. We observed some PAS‐stained tubular structures inside the tumor, which communicated with the native blood vessels and contained red blood cells. Specifically, the tube was formed by lowly differentiated tumor cells that were covered with PAS‐stained basement membrane. A previous study has indicated that the GBM cells for forming VM are "tumor stem‐like cells," which can differentiate to vascular endothelial‐like cells, promoting angiogenesis in tumor tissues.^8^ In this study, we found that the numbers of VM in human GBM tissues were significantly associated positively with disease relapse, but negatively with the PFS periods in 27 GBM patients. If further confirmed, the number of VM may be valuable for prognosis of GBM in the clinic.

LncRNA can regulate many biological processes by interacting with miRNA, transcription factors, and other molecules to affect the proliferation, apoptosis, invasion, migration, autophagy, VM, EMT, and other biological behaviors.^9^, ^10^ Their abnormal expression is associated with the development and progression of malignant tumors. Previous studies have shown that the lncRNA HULC expression is upregulated in liver cancers,^11^ colon cancer, ovarian cancer, and other tumor tissues and is associated positively with tumor size, TNM stage, chemotherapy resistance, and poor prognosis.^12^, ^13^ Hence, lncRNA HULC acts as an oncogenic factor to promote the progression of several types of malignant tumors. Our previous study has shown that lncRNA HULC expression is significantly upregulated in human GBM.^14^ The bioinformatic analysis indicated that patients with higher lncRNA HULC expression in their GBM tissues had a worse prognosis, and lncRNA HULC overexpression promoted the proliferation, migration, and invasion of human glioblastoma U87 cells by enhancing the HIF‐related PI3K/AKT/EGFR signaling.^15^ It is notable that the PI3K/AKT/EGFR signaling is also critical for GBM stem cell‐like cells to form VM as the PI3K activation can induce MMP14 expression and MMP2 maturation. The MMP2 is important for the construction of vascular basement membrane, and promote VM formation in solid GBM.^16^, ^17^ In this study, we explored the role for altered lncRNA HULC expression in the tubular formation and MMP expression in GBM cells after establishment of stable HULC overexpressing and silencing GBM cells. We found that compared with the controls, HULC overexpression significantly increased the numbers of tubular structures in both SHG44 and U87 cells while HULC silencing decreased their numbers. Similarly, HULC overexpression significantly upregulated MMP2 and MMP9 expression while HULC silencing downregulated their expression in both GBM cells in vitro and in vivo. Our data suggest that lncRNA HULC may promote VM by enhancing MMP2 and MMP9 expression in GBM. Such novel findings may provide new insights into the molecular pathogenesis of GBM and the lncRNA/MMP2/MMP9 may be new therapeutic targets for intervention of GBM progression.

Previous studies have shown that the signal pathways and transcription factors regulating the EMT process are important for VM, which suggests that VM is closely related to the EMT process. Twist1 overexpression enhances tubular formation and MMP expression in GBM cells, indicating that Twist1 can promote the formation of VM in tumor tissue.^18^, ^19^ Actually, the EMT process enhances the plasticity of tumor cells and extracellular matrix production for VM, and facilitates the formation of vascular basement membrane‐like structures.^20^ In this study, we found that lncRNA HULC overexpression enhanced the EMT process in GBM in vitro and in vivo. Evidently, lncRNA HULC overexpression decreased E‐cadherin expression, but increased N‐cadherin, vimentin, Snail, and Slug expression in both SHG44 and U87 cells. In contrast, lncRNA HULC silencing had opposite effects. More importantly, lncRNA HULC overexpression also enhanced the invasion of both SHG44 and U87 cells while lncRNA HULC silencing inhibited their invasion in vitro. It is possible that lncRNA HULC may enhance the HIP‐1α‐related PI3K/AKT/mTOR signaling, which cross‐talks with the Wnt/β‐catenin signaling to upregulate TGF‐β and Snail expression, promoting the EMT process in GBM cells.^21^, ^22^, ^23^ We are interested in further investigating the molecular mechanisms by which lncRNA HULC regulates the process of EMT and VM in GBM.

In summary, our data indicated that VM was associated with the progression and prognosis of GBM. We found that lncRNA HULC overexpression significantly enhanced the tubular formation and invasion of human GBM cells and promoted the growth of GBM tumors in mice while lncRNA HULC silencing had opposite effects. Furthermore, lncRNA HULC overexpression increased the EMT process and MMP expression while lncRNA silencing decreased them in GBM. Hence, lncRNA HULC may increase VM to promote the progression of GBM by enhancing the EMT process in GBM. Therefore, our findings may uncover a therapeutic target and help in understanding the molecular pathogenesis of GBM.

## CONFLICTS OF INTEREST

The authors state no conflicts of interest in this work.

## AUTHOR CONTRIBUTION

Tiantian Yin: Conducting the research and investigation process, performing the experiments, and writing the initial draft. Jing Wu: Data collection and presentation of the published work. Yuchen Hu: Formulation of overarching research goals and aims. Min Zhang: Provision of study materials, reagents, instrumentation, and other analysis tools. Jie He: Management and coordination responsibility for the research activity planning and execution.

## ETHICAL APPROVAL

The experimental protocols were approved by the Ethics Committee of University of Science and Technology of China (Ethical approval number: USTCACUC1801033). This paper has not been published elsewhere in whole or in part. All authors have read and approved the content, and agree to submit it for consideration for publication in your journal. There are no ethical/legal conflicts involved in the article.

## Data Availability

The data used to support the findings of this study are available from the corresponding author upon request.
